# Teaching and learning about dementia in UK medical schools: a national survey

**DOI:** 10.1186/1471-2318-13-29

**Published:** 2013-03-27

**Authors:** Ellen StClair Tullo, Adam L Gordon

**Affiliations:** 1 Newcastle NIHR Biomedical Research Centre in Ageing and Chronic Disease Newcastle University, Newcastle, UK; 2 Division of Rehabilitation and Ageing, School of Community Health Sciences, The University of Nottingham, Nottingham, UK

**Keywords:** Dementia, Education, Students, Medical, Curriculum

## Abstract

**Background:**

Dementia is an increasingly common condition and all doctors, in both primary and secondary care environments, must be prepared to competently manage patients with this condition. It is unclear whether medical education about dementia is currently fit for purpose. This project surveys and evaluates the nature of teaching and learning about dementia for medical students in the UK.

**Methods:**

Electronic questionnaire sent to UK medical schools.

**Results:**

23/31 medical schools responded. All provided some dementia-specific teaching but this focussed more on knowledge and skills than behaviours and attitudes. Only 80% of schools described formal assessment of dementia-specific learning outcomes. There was a widespread failure to adequately engage the multidisciplinary team, patients and carers in teaching, presenting students with a narrow view of the condition. However, some innovative approaches were also highlighted.

**Conclusions:**

Although all schools taught about dementia, the deficiencies identified represent a failure to sufficiently equip medical students to care for patients with dementia which, given the prevalence of the condition, does not adequately prepare them for work as doctors. Recommendations for improving undergraduate medical education about dementia are outlined.

## Background

Dementia is an increasingly common condition. In the UK, at least one quarter of acute hospital beds are occupied by patients with dementia, with admissions spread across a broad range of specialties [1,2]. Dementia care in the community is equally important, given that the majority of people with dementia live at home with their care primarily under the auspices of general practice [3]. The changing demographics of the UK population mean that the number of people living with dementia will rise for the foreseeable future – a projected 1 million by 2021 [2]. All doctors, both in primary and secondary care environments, must be prepared to provide competent care. However the 2009 UK *National Dementia Strategy* highlighted deficiencies in knowledge and skills of healthcare professionals caring for people with dementia [4]. A subsequent analysis of current education and training suggested that lack of education about dementia at the early stages of professional training “may be the most significant gap” [5].

It remains unclear how medical education about dementia is currently delivered, and how effective it is [6]. Against this background we set out to survey the current state of undergraduate medical education about dementia in order to clarify whether recent calls for mandatory inclusion of dementia-specific learning outcomes in undergraduate curricula [7] were being met, and identify what shortfalls in education, if any, were evident. We hoped also to identify exemplars of gold-standard teaching that could be shared.

## Methods

A questionnaire was developed and piloted by medical educators at two UK medical schools. The final questionnaire was published in electronic format using SurveyMonkey™ [8]. It contained both open and closed questions about teaching and learning on dementia – see Table 1 for a representation of the electronic questionnaire. There were free-text spaces for respondents to qualify their answers if necessary. The questionnaire was reviewed by the British Geriatrics Society’s (BGS) Education and Training Committee, and the study was endorsed by both the BGS and The Alzheimer’s Society before medical schools were approached.

**Table 1:** Questions included in the electronic questionnaire sent to medical schools


1. *Which medical school are you responding on behalf of?*	
2. *Is it possible to provide external access to the content of your curriculum?* Yes/No	
3. *How would you describe the structure of the curriculum at your medical school?* A:traditional*/*B:problem-based*/*C:integrated*/*D:spiral*/*E: accelerated/graduate*/*F:other (free text)	
4. *Which areas (courses/modules/clinical rotations) of your curriculum formally include dementia?* Free text	
5. *With reference to Q4, please supply curricular learning outcomes relevant to dementia*. Free text	
6. *Which methods are used to deliver teaching on dementia?* A:lecture/B:seminar*/*C:clinical placement*/*D:home/community visits*/*E:student assignment*/*F:case-studies*/*G:other (free text)	
7. *Which professionals are involved in the delivery of teaching on dementia?* A:old-age psychiatrist/B:GP/C:neurologist*/*D:geriatrician*/*E:mental health nurse*/*F:ethicist*/*G:other (free text)	
8. *Are curriculum outcomes on dementia formally assessed?* Yes/No	
9. *Has there been any attempt at you medical school to evaluate the impact of teaching about dementia e.g. student feedback, change in knowledge/skills/attitudes?* Yes (free text)/No/Don’t know	
10. *Have there been any changes, or plans for changes, to the curriculum in response to the National Dementia Strategy 2009?* Yes (free text)/No/Don’t know	
11. *Are people with dementia or their carers involved in the design or delivery of undergraduate medical education at your institution?* Yes (free text)/No/Don’t know	
12. *Are students guaranteed to interact with patients with dementia in the clinical environment before they graduate?* Yes*/*No*/*Don’t Know	
13. *Are students guaranteed to interact with carers of people with dementia with before they graduate?* Yes*/*No*/*Don’t know	
14. *Would you be prepared to be approached for ongoing consultation about the development of dementia-specific curriculum?* Yes/No*/*Maybe	
15. *Would you like a copy of the results of the survey*? Yes/No	
16. *Any further comments. (*Free text)	

The deans of all 31 UK medical schools were approached. A detailed description of these schools is beyond the remit of this article but is available at the website of the UK medical schools council (http://www.medschools.co.uk). Schools were contacted by email and letter, asking them to identify a respondent with an overview of the curriculum, who would be able to locate areas of the curriculum where dementia featured. The electronic survey was then sent to the nominated respondents, the majority of whom were academic clinicians with longstanding educational roles at the relevant medical school. Where initial approaches and reminders were unsuccessful, members of the BGS Education and Training committee representing regions with non-responding medical schools were contacted and invited to identify a respondent. As this was a survey of educational practices amongst UK universities, asking them to share information already in the public domain, no formal consent procedure was undertaken. Because responses were voluntary, participation was taken to imply consent to participate on behalf of the respondent.

Learning outcomes supplied by respondents were mapped to the three overarching themes outlined in *Tomorrow’s Doctors,* the national curriculum for UK medical undergraduates - the doctor as a scholar/scientist, practitioner and professional – which approximate a knowledge, skills and attitudes framework [9]. Following data collection and analysis a results summary was returned to the respondents for validation.

### Ethics

The project met the Newcastle University Preliminary ethical assessment guidelines, indicating that a Full University Ethics Committee review was not required.

## Results

Responses were received from 23/31 (74%) medical schools. One medical school declined to participate, two did not respond to any form of communication, and five initially agreed but did not subsequently complete the survey.

### Curriculum coverage

All responding medical schools identified at least one area of the curriculum where dementia featured. Six schools (26% of respondents) had made changes to their curriculum in response to the recommendations of the *National Dementia Strategy*, for example:

•The development of case-based discussions concerning patients with dementia

•Creation of a geriatric medicine module including specific teaching sessions on dementia

•Expansion of dementia-specific teaching to include end of life care, nutrition and advanced directives

Dementia education was incorporated into curricula in differing ways:

•Under the umbrella of “traditional” academic subjects such as pathology

•As part of a system-specific course such as a module concentrating on the sensory system

•As a condition within a specialty-specific module or rotation such as neurology, geriatrics or psychiatry

•In problem-based case studies focussing, for example, on a patient with confusion

•During the teaching of ethics, for example, issues surrounding consent and capacity

One medical school reported running a two–week dementia-specific course during which students interacted with patients with dementia and received teaching from professionals from a variety of disciplines in both primary and secondary care environments who contribute to the care of people with dementia.

### Learning outcomes

15 medical schools identified and provided examples of learning outcomes relevant to dementia. These outcomes covered different aspects of dementia care. Examples, mapped to the themes from *Tomorrow’s Doctors* are outlined in Table 2. Seven (47%) medical school curricula contained learning outcomes that mapped to all three domains.

**Table 2:** **Examples of dementia-specific learning outcomes mapped to the overarching outcomes of *Tomorrow’s Doctors***

**GMC outcome domain**	**Example learning outcomes**	**Number (%) of schools with relevant learning outcome**
The doctor as a scientist and scholar (knowledge)	Describe the brain changes that occur in dementia	11 (73%)
	Classify dementia according to cause	
The doctor as a practitioner (skills)	Take a relevant history, including an informant history	12 (80%)
	Perform a mental state examination including an examination of cognitive function.	
	Outline an investigation plan	
	Summarise the special considerations in prescribing psychotropic medications in the elderly	
The doctor as a professional (attitudes/behaviour)	Explain the medico-legal issues associated with dementia	10 (67%)
	Have an understanding of why care-giving for people with dementia is difficult	

### Teaching methods and assessment

The majority of medical schools used a range of teaching methods to deliver education about dementia (Table 3). 20 schools (87% of respondents) formally assessed students on learning outcomes specific to dementia. Two medical schools did not, and one could not be certain.

**Table 3:** Teaching methods to deliver education about dementia

**Teaching method**	**Number of responding schools (%)**
Clinical placement	22 (96%)
Lecture	20 (87%)
Seminar	18 (78%)
Case-studies	17 (74%)
Home/community visit	14 (61%)
Student assignment	7 (30%)
Problem-based learning	2 (9%)
E-learning podcast	1 (4%)

### Professionals involved in teaching

All responding schools used tutors from at least two disciplinary backgrounds. Old-age psychiatrists were the specialists most likely to be involved (96%), followed by geriatricians (87%), GPs (74%) and neurologists (57%). Other groups involved in teaching included mental health nurses (52%) and ethicists (43%).

### Evaluation of teaching

Seven schools had evaluated or were in the process of evaluating their teaching about dementia. Approaches to evaluation included written feedback from students, a survey to assess foundation doctors’ confidence in managing older people with dementia and measurement of student attitudes at the beginning and end of a psychiatry rotation.

### Involvement of people with dementia and their carers

Three schools directly involved people with dementia and their carers in the design and/or delivery of teaching. One school provided specific details of this as follows:

“*lay representatives from a local charity sit on the school’s curriculum group planning mental health teaching, and people with dementia and their carers are invited to participate in lectures and seminars for students.”*

17 (74%) medical schools were confident that medical students were guaranteed to interact with people with dementia in the clinical environment, although only nine (39%) were confident that medical students were guaranteed to interact with carers of people with dementia before graduation.

## Discussion

This study represents the most detailed description of undergraduate education around dementia in the UK undertaken to date. Its main findings are that all responding medical schools included dementia-related learning outcomes in their curricula but that there were widespread deficiencies in education relating to attitudes and behaviours, and a failure to ensure students had adequate exposure to patients with dementia and their carers.

Although dementia received universal coverage at some point in the curriculum, there appeared to be little consensus as to which aspects of dementia care should be core for medical education, with few schools including learning outcomes that mapped to all three themes in *Tomorrow’s Doctors*[9]. That teaching focussed more on knowledge and skills than behaviours and attitudes (Table 2) is perhaps unsurprising, given the acknowledged challenge of incorporating and assessing these domains in undergraduate curricula [10]; however it also represents a missed opportunity. The effect of education on attitudes towards dementia remains uncertain. However, there is a growing body of evidence that appropriately-targeted teaching interventions, for example, group discussion involving people with dementia and students, can positively influence students’ outlooks [11]. Reflecting on the needs of patients with dementia provides students with a prism through which they can consider diverse professional matters including ethics, patient dignity and multi-disciplinary teamwork. Curriculum planners should recognise dementia as an important opportunity to focus on generic professional development. Inclusion of topics previously identified as essential to good dementia care such as sensitive communication and a person-centred approach [12] should be viewed as central to professional development. It is possible that effective teaching about the skills and attitudes required to manage dementia would produce doctors better equipped to manage all of their patients, and not just those with dementia – this would be a legitimate focus of future research.

Given the emphasis of even lay media on the rapidly expanding population of patients with dementia, it is surprising that several medical schools were unable to guarantee that students will interact with people with dementia at all. Schools were even less confident that students would interact with the carers of people with dementia. Most teaching, meanwhile, was reported as being facilitated by doctors, particularly old age psychiatrists and geriatricians, indicating a missed opportunity to engage nurses and other members of the MDT in teaching. The lack of teaching delivered by nurses, allied healthcare professionals and lay carers may be a reflection of the practical difficulties of engaging non-clinicians in medical education. However, such an omission perpetuates the portrayal of a narrow, and potentially misleading, view of dementia, potentially compounding misunderstandings established through the lack of focus on behaviours and attitudes.

That fact that some medical schools do not assess dementia-oriented learning outcomes is an important finding. Assessment plays a pivotal role in learning. Ramsden [13] stated that, for many students, assessment is the curriculum—students focus their efforts on learning outcomes that they know are assessed. Biggs [14] proposed that students are more motivated if outcomes assessed map closely to those specified in the curriculum and taught during the course (‘curricular alignment’). Thus, a failure to assess core concepts in dementia may result in a failure to learn core concepts about dementia.

The survey highlighted notable examples of innovative approaches to teaching about dementia. One medical school included 2 week-long attachment in dementia care, where students are taught about the assessment and treatment of dementia in multi-disciplinary environments including community psychiatry, inpatient old age psychiatry, and acute medicine. Other innovative approaches directly sought the involvement of patients and carers in the design and delivery of teaching. One medical school involved the local branch of a dementia-specific charity in teaching, and provided students with an e-learning podcast relating to a carer’s experience of dementia. There are multiple potential benefits of involving patients and carers in teaching such as student satisfaction, improved student attitudes, and raised self-esteem of patients and carers who participate [15]. These benefits are likely to be true also of people with dementia and their carers.

### Limitations

The study has a number of limitations. All UK medical schools were not represented, although the response rate of 76% is high and allows some important generalisations to be made. The majority of data was collected with the help of only one representative at each institution. It is likely that some of these representatives may have had an inadequate overview of the undergraduate curriculum and that not all instances of dementia-specific teaching were reported. For medical schools with an integrated curriculum, it may have been difficult to extract dementia-specific learning outcomes from teaching domains such as medical ethics or chronic illness. A cross-sectional survey is only a snapshot and curricula are dynamic. It is likely that some of the curricula surveyed will already have changed since our work. Our survey was limited to dementia and, as such, we are unable to comment as to whether the deficiencies recognised extend to other common geriatric syndromes. Large national surveys considering the delivery of teaching across the totality of geriatric medicine have taken a more superficial overview, simply considering whether teaching in ageing-related topics was delivered and by whom [16]. Detailed survey work, similar to that undertaken here, would be required for other core topics in ageing to allow us to understand how best to allocate time and resources between dementia and other important areas.

## Conclusions

In centuries past, medical students in the UK learnt about rheumatic fever, syphilis and tuberculosis, as a reflection of the needs of the community that they served. Times have changed, and education about age-related diseases such as dementia should constitute the core of 21st century medical curricula, acting as an exemplar for professional development. It is clear that medical schools have recognised this, in principle at least. However the failures to focus adequately on behaviours and attitudes, to ensure contact with dementia patients and their carers and to adequately engage the multidisciplinary team in teaching demonstrate that insufficient thought has been given to how best to equip doctors to provide care to this rapidly expanding cohort.

We acknowledge that design, delivery and evaluation of teaching are complex processes that should be determined by local needs, however, we believe that there are immediate improvements that can be made to dementia-specific education at all UK medical schools. Whilst the survey relates most immediately to the UK, the increasing incidence of dementia globally [17] means that ongoing review of appropriate education and training for professionals caring for people with dementia is an international necessity [18]. In addition to the UK, mandatory inclusion of dementia in undergraduate medical curricula has similarly been recommended across Europe [19,20]. Based on the findings of the study, we make the following recommendations to medical educators in the UK, which may also be relevant to medical educators internationally, depending on local circumstances:

•Embed dementia as a core curriculum topic and ensure that dementia teaching covers all three themes from *Tomorrow’s Doctors*.

•Use dementia teaching as an opportunity to explore behaviours and attitudes to encourage professional development – for example through reflective teaching on person-centred care.

•Ensure that dementia-specific learning outcomes are assessed as well as taught.

•Involve the full multidisciplinary team in teaching.

•Involve people with dementia and their carers in teaching.

## Competing interests

The authors declare that they have no competing interests.

## Authors’ contributions

ET and AG designed the survey, ET collected survey data, ET and AG analysed survey data and drafted the manuscript. Both authors read and approved the final manuscript.

## Authors’ information

ET is a teaching and research fellow at NIHR Newcastle Biomedical Research Centre in Ageing. AG is a Consultant and Honorary Lecturer in Medicine of Older People, Division of Rehabilitation and Ageing, University of Nottingham.

## Pre-publication history

The pre-publication history for this paper can be accessed here:

http://www.biomedcentral.com/1471-2318/13/29/prepub

## References

[B1] Royal College of Psychiatrists Who cares wins. Improving the outcome for older people admitted to the general hospital. Guidelines for the development of liaison mental health services for older people 2005 London: Royal College of Psychiatrists

[B2] Alzheimer’s Society Dementia 2012: A National Challenge 2012 London http://www.alzheimers.org.uk/dementia2012

[B3] National Audit Office Improving services and support for people with dementia 2007 London: The Stationary Office

[B4] Department of Health Living well with dementia: A National Dementia Strategy 2009 London http://www.nao.org.uk/report/improving-services-and-support-for-people-with-dementia/

[B5] Department of Health and Skills for Care Working to support the implementation of the National Dementia Strategy project: Mapping existing accredited education/training and gap analysis report 2010 London: Department of Health http://www.skillsforhealth.org.uk/service-area/dementia/

[B6] Tullo E Allan L What should we be teaching medical students about dementia? Int Psychogeriatr 2011 23 1044 1050 10.1017/S1041610211000536 21457609

[B7] Royal College of Psychiatrists Report of the national audit of dementia care in general hospitals 2011 London: Royal College of Psychiatrists

[B8] SurveyMonkeyhttp://www.surveymonkey.com/

[B9] General Medical Council Tomorrow’s doctors: Outcomes and standards for undergraduate medical education 2009 London: General Medical Council

[B10] Stephenson AE Adshead LE Higgs RH The teaching of professional attitudes within UK medical schools: reported difficulties and good practice Med Educ 2006 40 1072 1080 10.1111/j.1365-2929.2006.02607.x 17054616

[B11] George DR Stuckey HL Dillon CF Whitehead MM Impact of participation in TimeSlips, a creative group-based storytelling program, on medical student attitudes toward persons with dementia: A qualitative study Gerontologist 2011 51 699 703 10.1093/geront/gnr035 21665958 PMC3247803

[B12] Department of Health and Skills for Care Core principles for supporting people with dementia. A guide to training the social care and health workforce 2011 London

[B13] Ramsden P Learning to Teach in Higher Education 1992 London: Routledge

[B14] Biggs J Enhancing teaching through constructive alignment High Educ 1996 32 347 364 10.1007/BF00138871

[B15] Towle A Bainbridge L Godolphin W Katz A Kline C Lown B Madularu I Solomon P Thistlethwaite J Active patient involvement in the education of health professionals Med Educ 2010 44 64 74 10.1111/j.1365-2923.2009.03530.x 20078757

[B16] Gordon AL Blundell AG Gladman JRF Masud T Are we teaching our students what they need to know about ageing? Results from the UK National Survey of Undergraduate Teaching in Ageing and Geriatric Medicine Age Ageing 2010 39 385 8 10.1093/ageing/afq011 20176711

[B17] Alzheimer’s Disease International World Alzheimer’s Report 2011 2011 London http://www.alz.co.uk/research/WorldAlzheimerReport2011.pdf

[B18] Doyle C International perspectives on dementia education, training and knowledge transfer Int Psychogeriatr 2009 21 Suppl 1 2 19288967 10.1017/S1041610209008734

[B19] Hasselbalch SG Baloyannis S Denislic M Dubois B Oertel W Rossor M Tsiskaridze A Waldemar G Education and training of European neurologists in dementia Eur J Neurol 2007 14 505 509 10.1111/j.1468-1331.2006.01679.x 17437608

[B20] Tsolaki M Papliagkas V Anogianakis G Bernabei R Emre M Frolich L Visser PJ Michel JP Pirttila T Olde Rikkert M Soininen H Soow T Vellas B Verhey F Winblad B European Alzheimer Disease Consortium Consensus statement on dementia education and training in Europe J Nutr Health Ageing 2010 14 131 135 10.1007/s12603-009-0238-z PMC12878970 20126961

